# 15q11.2 CNV affects cognitive, structural and functional correlates of dyslexia and dyscalculia

**DOI:** 10.1038/tp.2017.77

**Published:** 2017-04-25

**Authors:** M O Ulfarsson, G B Walters, O Gustafsson, S Steinberg, A Silva, O M Doyle, M Brammer, D F Gudbjartsson, S Arnarsdottir, G A Jonsdottir, R S Gisladottir, G Bjornsdottir, H Helgason, L M Ellingsen, J G Halldorsson, E Saemundsen, B Stefansdottir, L Jonsson, V K Eiriksdottir, G R Eiriksdottir, G H Johannesdottir, U Unnsteinsdottir, B Jonsdottir, B B Magnusdottir, P Sulem, U Thorsteinsdottir, E Sigurdsson, D Brandeis, A Meyer-Lindenberg, H Stefansson, K Stefansson

**Affiliations:** 1 deCODE Genetics/Amgen, Reykjavik, Iceland; 2 Faculty of Electrical and Computer Engineering, University of Iceland, Reykjavik, Iceland; 3 Cardiff University Brain Imaging Research Center, Cardiff University, Cardiff, UK; 4 Institute of Psychiatry, King's College, London, UK; 5 Faculty of Physical Sciences, University of Iceland, Reykjavik, Iceland; 6 Department of Psychiatry, Landspitali National University Hospital, Reykjavik, Iceland; 7 Faculty of Medicine, University of Iceland, Reykjavik, Iceland; 8 The State Diagnosis and Counselling Center, Kopavogur, Iceland; 9 Röntgen Domus, Reykjavik, Iceland; 10 School of Business, University of Reykjavik, Reykavik, Iceland; 11 Department of Child and Adolescent Psychiatry and Psychotherapy, Psychiatric Hospital, University of Zurich, Zurich, Switzerland; 12 Central Institute of Mental Health, University of Heidelberg Medical Faculty Mannheim, Mannheim, Germany

## Abstract

Several copy number variants have been associated with neuropsychiatric disorders and these variants have been shown to also influence cognitive abilities in carriers unaffected by psychiatric disorders. Previously, we associated the 15q11.2(BP1–BP2) deletion with specific learning disabilities and a larger corpus callosum. Here we investigate, in a much larger sample, the effect of the 15q11.2(BP1–BP2) deletion on cognitive, structural and functional correlates of dyslexia and dyscalculia. We report that the deletion confers greatest risk of the combined phenotype of dyslexia and dyscalculia. We also show that the deletion associates with a smaller left fusiform gyrus. Moreover, tailored functional magnetic resonance imaging experiments using phonological lexical decision and multiplication verification tasks demonstrate altered activation in the left fusiform and the left angular gyri in carriers. Thus, by using convergent evidence from neuropsychological testing, and structural and functional neuroimaging, we show that the 15q11.2(BP1–BP2) deletion affects cognitive, structural and functional correlates of both dyslexia and dyscalculia.

## Introduction

Specific learning disorders, such as dyslexia (DLX) and dyscalculia (DC), are challenging phenotypes to disentangle. DLX and DC refer to neurodevelopmental disorders manifested in learning difficulties with impairment in acquiring skills in reading and arithmetic, respectively, not due to intellectual disabilities or other developmental or neurological disorders.^1^ Despite being highly heritable, *h*^2^=0.52 for DLX and 0.61 for DC,^2^ genome-wide association studies have failed to uncover sequence variants conferring risk of these specific learning disorders.^3, 4^ Hence, larger genome-wide scans are needed to unravel how a confluence of rare and common sequence variants confer risk and which biological pathways are affected.

Prevalence of DLX and DC range from 4 to 7% depending on the criteria used.^5^ These learning disorders co-occur much more frequently than expected by chance; the comorbidity rate has been estimated as high as 40%.^6^ Although DLX and DC occur more often separate from each other, and largely distinct brain systems handle reading and mathematics, certain brain regions are important for both. Regions that have been associated with both disorders include the fusiform gyrus (BA37), which lies below the lingual and parahippocampal gyri and above the inferior temporal gyrus, as well as the angular gyrus (BA39), which is located in the posterior part of the inferior parietal lobe. The fusiform gyrus is thought to be an important structure for discriminating between and within categories of objects and includes the left hemisphere ‘visual word form area'. Dysfunction in these areas can lead to reading and/or math difficulties; the left fusiform gyrus has been shown to have less gray matter density and activation in individuals diagnosed with DLX.^7^ The angular gyrus^8^ has been shown to associate with high-level language and mathematical tasks, such as arithmetic fact retrieval.^9^

Some rare copy number variants (CNVs) are associated with neuropsychiatric disorders. Although little is known about how these high-impact variants confer risk of disease, they provide a biologically defined entry point for investigations into the mechanisms of brain function. These CNVs impact cognitive functions and learning and are probably the strongest identifiable factors contributing to the disease in affected carriers.^10, 11, 12^ An example is the 15q11.2(BP1–BP2) deletion that confers risk of neuropsychiatric disorders including specific learning difficulties. We have previously described the impact of the 15q11.2(BP1–BP2) deletion on cognitive abilities assessed by neuropsychological tests.^11^ Deletion carriers show modest impairments in most cognitive domains and the deletion confers high risk for DLX and DC. Here we investigate the effect of the 15q11.2(BP1–BP2) deletion on cognition, brain structures and functions of deletion carriers in a larger sample. Through neuropsychological testing, we establish that the cognitive profile of the deletion carriers is similar to the cognitive profile of the combined phenotype of dyslexia and dyscalculia. By using magnetic resonance imaging (MRI) and functional MRI (fMRI), we show that the deletion affects structural and functional correlates of DLX and DC.

Phenotypic heterogeneity, caused by many different biochemical perturbations, complicates the search for sequence variants conferring risk of DLX and DC. Here we focus on the impact conferred by the 15q11.2(BP1–BP2) deletion and show that the sequence variant confers risks of both DLX and DC and the carriers have cognitive, structural and functional aberrations that are considered to be correlates of both conditions.

## Materials and methods

### Participants

Subjects carrying the 15q11.2(BP1–BP2) deletion, and controls not carrying CNVs associated with psychiatric disorders (NoCNV), were recruited from a large genotyped sample of approximately 160 000 subjects representing half of the Icelandic population. Only subjects aged between 18 and 65 were included in this study. Subjects were excluded: if they had ICD-10 or DSM-IV diagnoses for schizophrenia, schizoaffective or bipolar disorder; if they were diagnosed with autism, intellectual disability or developmental delay at the State Diagnostic and Counselling Centre of Iceland serving children and adolescents with a disability; if they met psychoses criteria on the MINI^13^ interview; if they were diagnosed with schizophrenia, schizoaffective, bipolar disorder, autism, intellectual disability or developmental delay according to self-reports (or reports from parents); if they were using antipsychotic medication. In the neuroimaging experiment, we used a subset of the NoCNV group of subjects without any large CNVs (PopCtrl). Supplementary Table 1 shows the population characteristics for subjects participating in the neuroimaging experiments. All the participants signed informed consent approved by the National Bioethics Committee of Iceland.

### Cognitive phenotyping

**Figure 1:**
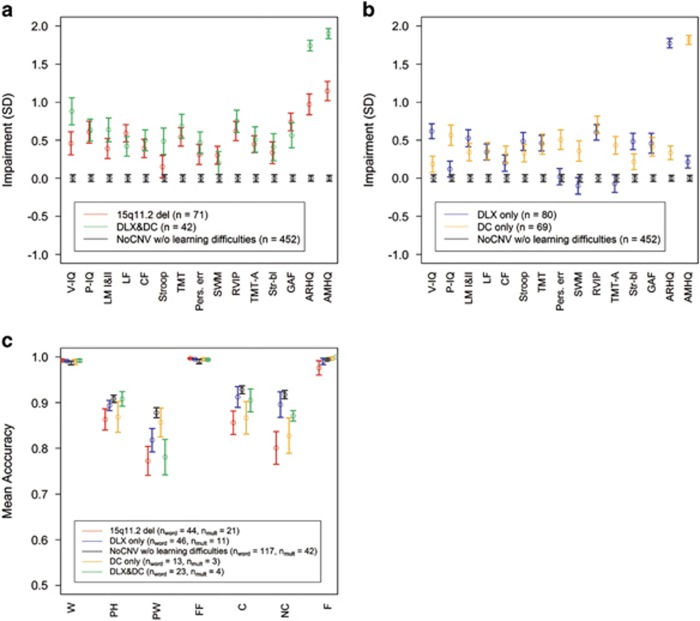
Association of the 15q11.2(BP1–BP2) deletion group and subgroups of NoCNV with cognitive traits, GAF, ARHQ, AMHQ and functional MRI test scores. (**a**) Average standardized scores for 15q11.2(BP1–BP2) deletion, DLX&DC and NoCNV without (w/o) learning difficulties. (**b**) Average standardized scores for DLXonly, DConly and NoCNV w/o learning difficulties. (**c**) Mean accuracy for fMRI phonological lexical decision tasks (words) and multiplication verification tasks (mult) for 15q11.2(BP1–BP2) deletion, DLXonly, DConly, DLX&DC and NoCNV. The tests are verbal IQ (V-IQ); performance IQ (P-IQ); logical memory I and II (LM I and II); letter fluency (LF); category fluency (CF); Stroop (the difference between the time it takes to name the color of the ink of a word that is actually the name of another color and to name the color of colorpads); trail making test (TMT), TMT trail B–TMT trail A; perseverative errors in the Wisconsin card sorting test (Pers. Err); spatial working memory (SWM); rapid visual information processing (RVIP); TMT trail A (TMT-A); Str-bl (Stroop: time it takes to read the names of colors written in black ink); global assessment of function (GAF); adult reading history questionnaire (ARHQ); adult mathematical history questionnaire (AMHQ). Word experiment: orthographic familiar forms of Icelandic nouns (W); phonologically correct but orthographically unfamiliar forms of the same word (PH); phonologically and orthographically unfamiliar forms (PW); false fonts (FF). Multiplication experiment: correct equation (C); incorrect equation (NC); false equation (F). See previous study^11^ for more information about tests in **a** and **b**, and the functional MRI section below for more information about tests in **c**. Error bars represent standard error. Impairment is in s.d. units. The sample size given in the figure legend for **a** and **b** refer to the number of subjects with available scores. Some individual scores are missing. The sample size for each test is given in Supplementary Table 2. CNV, copy number variation; DC, dyscalculic; DConly, dyscalculic but not dyslexic; DLX, dyslexic; DLX&DC, dyslexic and dyscalculic; DLXonly, dyslexic but not dyscalculic; IQ, intelligence quotient; MRI, magnetic resonance imaging.

A total of 71 subjects carrying the 15q11.2(BP1–BP2) deletion in the absence of a schizophrenia, bipolar disorder, autism or intellectual disability diagnosis were recruited along with 643 controls not carrying CNVs associated with psychiatric disorders (NoCNV). Participants were assessed with a battery of neuropsychological tests measuring cognitive traits, the global assessment of functioning scale and self-reported questionnaires on reading (adult reading history questionnaire, ARHQ) and mathematics (adult mathematical history questionnaire, AMHQ). Psychologists and others phenotyping the study subjects were blind to the genotype. Large lists of CNV-carriers and non-carriers were sent to a clinic overseeing the phenotyping. Identifiers were encrypted and sent back to researchers working on the genetic data. A detailed definition of the tests and questionnaires is given in a previous study (see also Figure 1).^11^ Supplementary Table 2 shows the sample sizes for the tests and questionnaires. To investigate the deletion group with respect to reading and math, the NoCNV group was further separated into six subgroups defined by using a score greater than 0.43 on the ARHQ,^14^ and a score greater than 12 on the AMHQ^11^ as a surrogate for dyslexia and dyscalculia, respectively. These subgroups contain (i) 80 dyslexic but not dyscalculic (DLXonly), (ii) 69 dyscalculic but not dyslexic (DConly), (iii) 42 dyslexic and dyscalculic (DLX&DC), (iv) 452 NoCNV without specific learning difficulties, (v) 123 dyslexic (without regard to the dyscalculic status) (DLX), (vi) 111 dyscalculic (without regard to the dyslexic status). Supplementary Table 3 presents the carrier status and the number of individuals in each subgroup.

### Statistical analysis of cognitive traits

The scores from each cognitive test or questionnaire were inverse normally transformed and then adjusted for gender, age at testing and where indicated, intelligence quotient (IQ) based on data from controls only. The scores were shifted and scaled so that controls had a mean of 0 and a standard deviation of 1, and also arranged so that higher scores indicated greater impairment. Fisher's exact test and the DLX and DC subgroup information in Supplementary Table 3 was used to estimate the deletions' risk of DLX, DC and DLX&DC. A result was judged as significant when the *P*-value was less than 0.05 Bonferroni corrected for the number of cognitive tests or questionnaires.

### Structural MRI data acquisition

The subjects listed in Supplementary Table 1 were scanned using an MRI scanner (1.5 T Philips Achieva, Philips Medical Systems, Eindhoven, Netherlands). The scans were performed with a three-dimensional fast T1-weighted gradient echo sequence (TR=8.6 ms, TE=4 ms, flip angle=8 degrees, slice thickness 1.2 mm, matrix=192 × 192, field of view=240 × 240 mm). This MRI protocol was selected as it yields good contrast between white matter (WM), gray matter (GM) and cerebrospinal fluid. Quality control consisted of visual inspection as well as a test of homogeneity of the image covariance, which is a part of the voxel-based morphometry (VBM) protocol described below. A total of 716 participants were scanned with 707 subjects passing quality checks.

### Voxel-based morphometry

By using VBM,^15, 16^ we analyzed the allele dose-dependent effect of CNV on white and gray matter tissue across subjects, while controlling for age and gender. VBM is a technique that allows investigation of regional differences in the brain anatomy. In this study, the T1-weighted structural brain MRIs were analyzed using VBM as implemented by the VBM8 software (http:\dbm.neuro.uni-jena.de) (version r351), which is integrated into the SPM8 software (Wellcome Department of Cognitive Neurology, Institute of Neurology, London, UK, (http://www.fil.ion.ucl.ac.uk/spm)) implemented in MATLAB R2013b (Mathworks, Sherborn, MA, USA). Briefly, each T1-weighted structural brain MRI is tissue-segmented into WM, GM and cerebrospinal fluid images, which are then registered to the MNI (Montreal Neurological Institute)^17^ space using an affine transformation. After the tissue segmentation step, a spatial normalization step was performed where each tissue segment is brought into a common stereotactic space. This step uses the DARTEL algorithm^18^ and a brain template derived from 550 healthy control subjects in the IXI-database (http://www.brain-development.org). Finally, the maps from the normalization step were modulated, that is, intensity-corrected for local volume changes during the spatial normalization. To accommodate for noise and registration errors, the modulated maps were smoothed with a 12 mm full-width half-maximum Gaussian filter.

### Statistical analysis of the structural brain MRI data

Multiple regression analysis was used to test carrier status effects on brain volume on a voxel-wise basis using SPM8. We examined regions throughout the entirety of the brain for volume differences using the model:


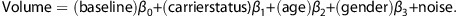


The carrier status was modeled by using a regressor coding deletion as 0, PopCTRL as 1 and duplication as 2. Age and gender were added in the statistical model as covariates of no interest. Furthermore, we inspected the effects of adding intracranial volume as a covariate of no interest but it had minor effect on the results and did not change the conclusions. The carrier status effects were tested using one-sided *t*-tests and the voxel-wise effects on gray and white matter volume were reported as significant when a whole-brain family-wise error-corrected *P*-value was less than 0.05. Owing to the intrinsic spatial smoothness of the data, the Bonferroni correction procedure is overly conservative. Therefore, we use the random field theory^19, 20^ correction method as implemented in the SPM8 toolbox to correct for multiple comparisons.

### Functional MRI

The subjects listed in Supplementary Table 1 participated in two fMRI experiments, the first involving a phonological lexical decision task (word experiment), and the second involving a multiplication verification task (multiplication experiment).

### Functional MRI acquisition

The data were collected using a 1.5 T Philips Achieva MRI scanner. Two hundred and sixty-five volumes (185 volumes in the short version; see below) and 215 volumes were acquired for the words and multiplication experiments, respectively. In both experiments, 28 axial brain slices were acquired using an echo planar imaging pulse sequence (TR=3 s; TE=55 ms; image matrix=64 × 64; voxel size=3.75 × 3.75 × 5 mm^3^; flip angle=90° slice order=ascending, sequential; coverage=whole brain).

### fMRI word experiment

During the fMRI scanning, the participants were asked to decide whether a visually presented letter string sounded like a real word or not. The experimental design follows a previous fMRI study^21^ closely with the main difference being that it was modified for Icelandic native speakers. There were two versions of the experiment: the long version and the short version. In the long version, there were 176 stimuli, lasting one second each, consisting of four types: 44 orthographically familiar forms of Icelandic nouns (W), 44 pseudohomophones that were phonologically correct but an orthographically unfamiliar form of the same word (PH), phonologically and orthographically unfamiliar forms (PW) and 44 false fonts (FF). In addition, there were 59 null events where only the fixation cross was presented. In the short version, there were 120 stimuli, 30 for each stimuli type (W, PH, PW and FF) and 43 null events. Subjects were excluded from the analysis if they (i) exceeded *a priori* maximum movement criterion (±3 mm translation or ±3° rotation), (ii) performed poorly in the phonological lexical decision task (<60% accuracy in one or more stimuli type) or (iii) had poor quality MR data. A total of 337 participants were scanned with 284 subjects passing quality checks.

### fMRI multiplication paradigm

During fMRI scanning, the participants were asked to verify whether a visually presented multiplication equation was correct or not. There were 132 stimuli consisting of three types: 44 correct (C) multiplication equations, for example, 5 × 9=45; 44 incorrect (NC) multiplication equations, for example, 6 × 6=21; 44 false (F) equations, for example, 9 q 4=lv. The correct equations were selected from the 10 × 10 multiplication table. Subjects were excluded from the analysis according to the same exclusion criteria as in the word experiment. A total of 117 participants were scanned with 110 subjects passing quality checks.

### Statistical analysis of the fMRI data

The data were analyzed using SPM12. The statistical analysis described here is only for the word experiment, the analysis was similarly performed for the multiplication test. The data were first realigned to the mean, using a rigid model, followed by a slice timing correction. After that, the mean image of each echo planar imaging time series was spatially normalized to the SPM's MNI152 template. The images were then spatially resampled to 2 × 2 × 2 mm^3^. Finally, the images were spatially smoothed using a 9 mm full-width at half-maximum Gaussian kernel.

A two-stage model was used for statistical analysis assuming a mixed-effect design. In the first stage, event types representing the correct responses for each stimuli type (W, PH, PW and FF) were modeled using the standard SPM hemodynamic function with its temporal derivative. The incorrect stimuli responses were modeled the same way and were also included in the model. The model also included six movement regressors from the realignment step.

In the second stage, the following contrasts W, PH, PW, FF, W vs FF, FF vs W, PH vs W, W vs PH, W vs PW and PW vs W, were tested using a one-sided *t*-test using the following multiple regression model:


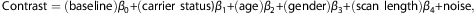


where the carrier status was modeled using a regressor coding deletion as 0, PopCTRL as 1 and duplication as 2, and the scan length was modeled by coding the long-word version with 1 and the short-word version with 2; scan length was not included as a covariate in the regression model for the multiplication test. Given our *a priori* hypothesis and the brain-wide significant results from the structural analysis, we constructed a mask using the Automatic Anatomic Labeling atlas^22^ consisting of the left fusiform gyrus, left parahippocampal gyrus, inferior parietial lobule, angular gyrus, and supramarginal gyrus. We reported voxel-wise carrier status effects on the contrasts significant when the family-wise error-corrected^19, 20^ (within this mask) *P*-value was less than 0.05. In the case of the multiplication test, the contrasts C, F, F vs C, C vs F, NC, NC vs C, C vs NC, F vs NC and NC vs F were analysed.

### Correlations between the brain imaging and the cognitive phenotypes

The Pearson's correlation measure between each of the cognitive tests/questionnaire score in Figure 1 and the brain imaging phenotype in Table 1 for both the NoCNV group and the 15q11.2(BP1–BP2) deletion group was computed. All the scores were corrected for gender and age before the correlation was computed. The brain phenotypes were the raw volume scores (for the VBM data) and contrast scores (for the fMRI data) at the locations indicated in Table 1. A result was judged as significant when the *P*-value was less than 0.05 Bonferroni corrected for the number of correlations computed.

## Results

### Neuropsychology

The same neuropsychiatric CNV often confers risk of a range of neurodevelopmental phenotypes including schizophrenia, autism, intellectual disability, attention deficit hyperactivity disorder and epilepsy. The 15q11.2(BP1–BP2) deletion has been associated with schizophrenia as well as specific learning disorders.^11^

Our previous results show that 15q11.2(BP1–BP2) deletion carriers unaffected by neuropsychiatric disorders do perform worse on neuropsychological tests and are more likely to suffer from specific learning disorders than population controls.^11^ Hence, when learning difficulties are considered, the deletion is likely to be fully penetrant although the expressivity may vary substantially. The deletion confers high risk of DLX (odds ratio=3.0, *P*=2.2 × 10^−4^) and DC (odds ratio=3.4, *P* =4.9 × 10^−5^) when using a score greater than 0.43 on the ARHQ^14^ and a score greater than 12 on the AMHQ^11^ as a surrogate for dyslexia and dyscalculia, respectively. The deletion confers a greater risk when considering the comorbid phenotype DLX&DC (odds ratio=4.4, *P*=1.3 × 10^−4^).

Considering all the tests in Figure 1a except for the ARHQ and the AMHQ tests, it can be seen that the deletion group and the DLX&DC group have a similar profile (Spearman's correlation between mean profile scores=0.58, *P*=0.042). The impairments (again excluding the ARHQ and the AMHQ tests) present in the DLXonly, and the DConly phenotypes combine additively to produce the impairments in the DLX&DC phenotype (Spearman's correlation between the sum of the DLXonly and DConly means and the DLX&DC means =0.63, *P*=0.023). The DLXonly and DConly groups (Figure 1b) clearly have different profiles, especially with regards to the IQ scores, perseverative errors in the Wisconsin card sorting test, spatial working memory and trail making test A.

The deletion group was compared with the combined group of DLXonly, DConly, DLX&DC and NoCNV without learning difficulties. The largest impairment is observed on the ARHQ (0.58 s.d., *P*=1.5 × 10^−4^) and the AMHQ scores (0.75 s.d., *P*=7.4 × 10^−7^). However, the deletion group also shows impairment on other scores. When the scores are corrected for performance IQ and verbal IQ, the impairments on ARHQ (0.44 s.d., *P*=0.0044), and AMHQ (0.64 s.d., *P*=3.5 × 10^−5^) remain, whereas the impairments measured by the other scores are not significant compared with a Bonferroni threshold of *P*=0.05/30=0.0017 accounting for the 15 tests with and without the IQ adjustment.

The deletion group shows impairments in reading and mathematics, based on the phonological lexical decision task (PW) and the multiplication task (NC; see Figure 1c for a definition) used to assess how well carriers recognize words and understand mathematical equations. The deletion carriers perform worse on both tests than controls (Figure 1c).

Subjects carrying 15q11.2(BP1–BP2) duplications were also investigated with respect to the aforementioned tests. No significant impairments in the 15 tests presented in Figures 1a and b were detected in the 15q11.2(BP1–BP2) duplication carriers as compared with the rest of the NoCNV group.

### Structural MRI phenotypes

We obtained structural brain MRI of 51 carriers of the 15q11.2(BP1–BP2) deletion not diagnosed with defined neuropsychiatric disorders, 104 carriers of the reciprocal duplication and 552 controls not carrying CNVs associated with psychiatric disorders and without large CNVs (PopCtrl).

**Figure 2:**
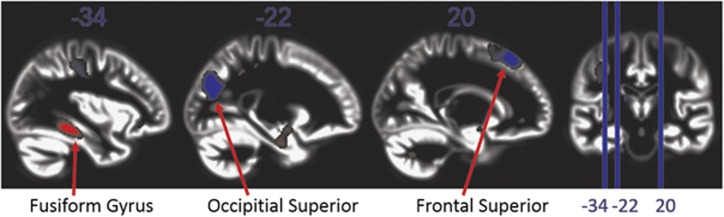
The effect of the CNV carrier status (deletion, PopCtrl, duplication) on gray matter volume difference. The *T*-scores for the CNV carrier status are displayed where findings are highlighted in red or blue if *P*<0.001 with red indicating less gray matter volume for the 15q11.2(BP1–BP2) deletion carriers, and blue indicating greater volume. The first three figures are sagittal slices while the last figure shows a coronal slice where the location of the sagittal slices are denoted by vertical lines. CNV, copy number variation.

**Figure 3:**
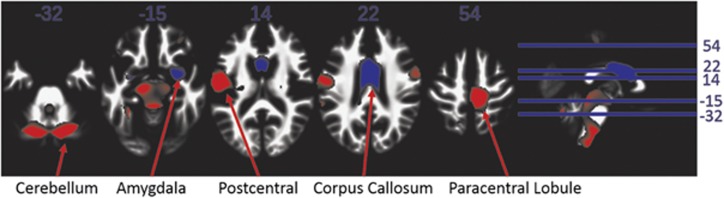
The effect of the CNV carrier status (deletion, PopCtrl, duplication) on white matter volume change. The *T*-scores for the CNV carrier status are displayed where findings are highlighted in red or blue if *P*<0.001 with red indicating less white matter volume for the 15q11.2(BP1–BP2) deletion carriers, and blue indicating greater volume. The first five images are axial slices (inferior to superior). The rightmost image shows the locations of the axial slices on a sagittal view. CNV, copy number variation.

We examined regions throughout the entirety of the brain and analysed the result using whole-brain family-wise error multiple comparison correction. For both GM and WM, the carriers of a deletion vs duplication showed mirrored effects, that is, the deletion carriers show opposite changes to the duplication carriers (Supplementary Figures 1 and 2). The 15q11.2(BP1–BP2) deletion carriers have less GM volume in the left fusiform gyrus extending into the parahippocampal gyrus, and greater GM volume in the superior occipital gyrus (which is a part of the visual association area) and the superior frontal regions (Figure 2 and Table 1). The area in the superior frontal gyri has been implicated in visual attention.^23^ The deletion carriers have less WM volume in the right cerebellum, the right paracentral lobule and the left superior temporal lobe. On the other hand, the deletion carriers have greater WM volume in the anterior corpus callosum and the right amygdala (Figure 3 and Table 1). We observed a significant interaction between carrier status of the CNV and gender in the right caudate nucleus (Supplementary Figure 3), that is, the female deletion carriers have greater GM volume than female duplication carriers, and on the other hand, male deletion carriers have less GM volume than the male duplication carriers (Supplementary Figure 4).

### Functional MRI phenotypes

Based on our *a priori* hypothesis, we performed two fMRI experiments: a phonological lexical decision task (word experiment) and a multiplication verification task (multiplication experiment). Given the results of the structural analysis, we restricted our fMRI analysis to the left occipito-temporal cortex and the left parietal lobe.

A total of twenty nine 15q11.2(BP1–BP2) deletion carriers, 191 PopCtrl subjects and 60 duplication carriers took part in the word experiment. The participants were asked to decide whether a visually presented letter string sounded like a real word or not. There were four types of letter strings: (i) orthographically familiar forms of Icelandic nouns (W), (ii) pseudohomophones that were phonologically correct but orthographically unfamiliar forms of the same words (PH), (iii) pseudowords that were phonologically and orthographically unfamiliar forms (PW) and (iv) false fonts (FF). The experimental design follows that of van der Mark *et al.,*^21^ with the main difference being the translation to Icelandic.

**Figure 4:**
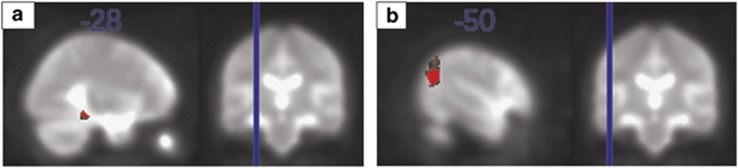
Carrier status-dependent functional difference. (**a**) The word experiment. A sagittal slice (left), *x*=−28, showing the location, MNI=(−28, −36, −14) of significant carrier status effect on the contrast PW vs W. (**b**) The multiplication experiment. A sagittal slice (left), *x*=−50, showing the location, MNI=(−50, −66, 24), of significant carrier status effect on the contrast C vs F. The right images on both **a** and **b** show the location of the sagittal slice on a coronal view. C, correct equation; F, false equation; MNI, Montreal Neurological Institute; PW, phonologically and orthographically unfamiliar forms; W, orthographic familiar forms of Icelandic nouns.

As with the structural MRI results, the effect of deletion vs duplication carrier status on brain volumes was mirrored (Supplementary Figure 5). The results show that the deletion carriers have less PW vs W contrast in the left fusiform gyrus (Figure 4, Table 1). A previous report demonstrated the presence of phonological and orthographic familiarity effects in non-dyslexic children. Non-dyslexic children showed higher activation for unfamiliar (PH and PW) rather than familiar (W) word-forms, whereas this effect was absent in children with dyslexia.^21^ The results presented here are in line with this, showing that the phonological/orthographic familiarity effect is decreased in the left fusiform gyrus of deletion carriers.

The multiplication experiment was performed on 18 deletion carriers, 40 PopCTRL and 52 duplication carriers. They were asked to determine whether a visually presented multiplication equation was correct or not. The equations were either correct (C), incorrect (NC) or false (F).

The 15q11.2(BP1–-BP2) deletion carriers have less C vs F contrast in the left angular gyrus (Figure 4, Table 1). It has been proposed that the left angular gyrus supports the retrieval of mathematical facts such as the multiplication table^24^ and also the usage of previously learned facts.^25^

### Correlations between the brain imaging and the cognitive phenotypes

The Pearson's correlation measure between each of the cognitive tests/questionnaire scores in Figure 1 and the brain imaging phenotypes in Table 1 for both the NoCNV group and the 15q11.2(BP1–BP2) deletion group was computed (Supplementary Tables 6 and 8). There are significant correlations within the cognitive tests/questionnaire scores and also within the brain imaging phenotypes. But no significant correlation between the brain imaging phenotypes and the cognitive tests/questionnaire scores. The failure to detect significant association between the cognitive tests/questionnaire scores and the brain imaging phenotypes could, at least in part, be explained by the loss of statistical power due to lower sample size for the intersection of the cognitive tests/questionnaire scores and brain phenotypes than for them separately (Supplementary Tables 7 and 9).

## Discussion

It has been argued that a complex set of impairments in brain function account for comorbidity of DLX and DC.^6^ Here we demonstrate that the same variant confers risk of both DLX and DC. It is, however, important to keep in mind that this variant encompasses several genes. Haploinsufficiency of the genes affected by the 15q11.2(BP1–BP2) deletion impacts both cognitive traits and brain structure in a pattern consistent with the cognitive, structural and functional correlates of DLX and DC.

The results show that the cognitive profile of the subjects carrying the 15q11.2(BP1–BP2) deletion resembles the cognitive profile of the DLX&DC subjects and, even after correcting for IQ, associates with the ARHQ and AMHQ scores. As the neuropsychological profiles of the DLXonly and DConly groups are clearly different and only when combined produce the impairments in the DLX&DC phenotype, it can be inferred that impairments in DLX and DC are additive, suggesting that the cognitive processes involved in DLX and DC are largely independent. However, the DLX and the DC phenotypes are clearly not independent since that would mean (assuming 7% prevalence rate of DLX and DC) that the prevalence of the comorbid phenotype would be 0.49%, which is not the case. This indicates that some unknown factor affects both the DLX and the DC cognitive processes in the DLX&DC phenotype. The neuroimaging data show that the 15q11.2(BP1–BP2) deletion affects both gray and white matter structures in the brain and is associated with specific changes in function relative to controls. In particular, the deletion affects the left fusiform gyrus and the left angular gyrus, brain structures that have been associated with both DLX and DC.

The lower GM volume in the left fusiform gyrus of the deletion carriers is of particular interest as it has been reported that this region has a major role in reading and mathematical processing.^26^ The fusiform gyrus is a part of the ventral temporal cortex,^27^ and is generally thought to be a key structure for high-level visual processing including face perception,^28^ reading^29^ and object recognition.^30^ Alterations in this region have also been associated with DLX in both structural,^31, 32, 33^ and functional studies,^21, 34, 35^ as well as with DC^36^ in morphometry and tractography studies. A recent meta-analysis of brain dysfunction in both DLX children and adults noted that the fusiform gyrus was the only brain region affected in both the groups.^24^

The decreased WM volume observed in the temporal lobe and cerebellum, and the greater WM volume in the corpus callosum replicate previous findings.^11, 37^ Overall, we note that the WM findings are stronger than the GM findings.

As the left fusiform gyrus is thought to support skilled and fluid reading, and the left angular gyrus to support retrieval of mathematical facts such as the multiplication table, the structural and functional alterations in those areas may be the cause of the specific learning disorders found in the deletion carriers as some abnormalities predate literacy^38^ and numeracy, but may also reflect a lack of reorganization due to emerging literacy^39, 40^ and/or numeracy. Although caudate GM volume alterations in DLX have been described previously,^41^ the interaction with gender is a novel finding and may reflect differences in articulatory compensation.^42^ The finding of decreased WM in the cerebellum could lend support to the cerebellar deficit theory,^43^ which states that dyslexia is characterized by a general cerebellar abnormality resulting in impaired ability to perform tasks automatically thereby negatively affecting language and reading. Overall, the data support that the 15q11.2(BP1–BP2) CNV maps to a multifocal neurobiological profile and the implicated structures fit well with those identified in studies on reading and/or math problems.

The 15q11.2(BP1–BP2) CNV shows an allele dose-dependent (mirrored) effect on both the structure and function of the human brain, that is, duplication carriers show reciprocal changes in exactly the same brain regions as the deletion. However, this was not observed for the cognitive traits. Although the deletion negatively impacts performance on cognitive tests, the duplication carriers performed on par with controls. A similar asymmetry between neuroimaging and cognitive phenotypes have been reported^44, 45^ for the 16p11.2 CNV where the cognitive performance is negatively impacted by both the deletion and the reciprocal duplication.

The BP1–BP2 region, spanning approximately 500 kb, contains four highly conserved, non-imprinted genes: *TUBGCP5*, *NIPA1*, *NIPA2* and *CYFIP1*. *NIPA1*, *NIPA2* and *CYFIP1* are highly expressed widely in the central nervous system, while *TUBGCP5* is highly expressed in the subthalamic nucleus.^46^ Yoon *et al.*^46^ took a multifaceted approach to investigate why the 15q11.2(BP1–BP2) deletion confers risk of neuropsychiatric disorders. They used human iPSC-derived neural progenitors carrying the deletion and noticed deficits in adherens junctions and apical polarity. They claim that these results from haploinsufficiency of *CYFIP1* encoding a subunit of the WAVE complex. They also demonstrated that in the developing mouse cortex, deficiency in *CYFIP1* and WAVE signaling similarly affects radial glial cells, leading to their ectopic localization outside of the ventricular zone.^46^ Bozdagi *et al.*^47^ furthermore reported that haploinsuffiency of *CYFIP1* produces fragile X-like phenotypes in mice. Thus, haploinsuffiency of *CYFIP1* may contribute to the neurodevelopmental origins of the disorders associated with the 15q11.2(BP1–BP2) deletion.^10, 46, 48^

This study adds to the emerging understanding of the impact conferred by the 15q11.2(BP1–BP2) deletion on brain structure and function. The deletion confers high risk of the DLX&DC phenotype (odds ratio=4.4, *P*=1.4 × 10^−4^), and the results demonstrate significant volume changes in WM and GM brain structures in addition to a decrease in brain activation in regions important for reading and arithmetic. Overall, our findings shed light on the role of this CNV in typical and atypical brain development. The deletion allele impacts cognitive function and learning and is probably the strongest factor contributing to the DLX&DC phenotype in the affected carriers.

Brain structure is largely shaped by sequence variants that exert lasting influences on its function and several common variants have been associated with subcortical structures.^49, 50^ The confluence of common variants predicting subcortical structures does, however, not predispose to brain diseases like schizophrenia.^51^ Thus, although brain structure volumes show high heritability, there may not be a direct correlation with diseases. Hence, although subcortical volumes may differentiate patients from controls, the explanation may not necessarily be rooted in their genomes. Many brain disorders are heterogeneous groups of disorders at the level of genetic etiology and clinical presentation. Through high-impact variants, the relationship between genotype and phenotype may be disentangled, which, in turn, may help determine which brain phenotypes, associated with a disease, are a cause or consequence of the disease. For instance, larger putamen and pallidum volumes associate with duration of illness in schizophrenia,^51^ a consequence of the disease that can be combatted with antipsychotic drugs.

Here we have demonstrated by using convergent evidence from neuropsychological testing and structural and functional neuroimaging that a high-impact sequence variant provides insight into the causes of variability in human brain structure and function. Although the 15q11.2 CNV alleles confer mirror effects on both brain structure and function, only the deletion affects cognition with large effect. Sequence variants influencing brain structures may reveal new biological mechanisms underlying cognition and neuropsychiatric illness.

## Figures and Tables

**Table 1:** Carrier status-dependent functional, gray and white matter volume changes

	*Hemisphere*	*MNI coordinates* *(*x*,*y*,*z*)*	*Effect (%)* *95% CI*	P*-value* *(corrected)*	*Brodmann* *area*
*sMRI: gray matter*					
Fusiform gyrus	Left	(−35, −36, −15)	+3.0 (2.9, 3.1)	0.045	BA37
Superior occipital	Left	(−22, −78, 24)	−4.8 (−5.0, −4.6)	0.016	BA19
Superior frontal	Right	(20, 30, 52)	−5.0 (−5.2, 4.8)	0.016	BA8
					
*sMRI: white matter*					
Cerebellum cruz 1	Right	(28, −72, −32)	+7.7 (7.6, 7.9)	6.84 × 10^−5^	
Paracentral lobule	Right	(10, −30, 54)	+4.6 (4.5, 4.7)	6.93 × 10^−4^	
Superior temporal	Left	(−52, −12, 13)	+5.0 (4.5, 5.1)	1.94 × 10^−3^	
Anterior corpus callosum	NA	(4, 0, 22)	−4.6 (−4.7, −4.5)	6.84 × 10^−4^	
Amygdala	Right	(26, 2, −17)	−4.7 (−4.8, −4.6)	5.57 × 10^−3^	
					
*fMRI word paradigm: PW vs W*					
Fusiform gyrus	Left	(−28, −36, −14)	+68.2 (63.7, 72.8)	0.007^a^	BA37
					
*fMRI multiplication paradigm: C vs F*					
Angular gyrus	Left	(−50, −66, 24)	+87.2 (80.8, 93.8)	2.08 × 10^−4^^a^	BA39

Abbreviations: C, correct equation; CI, confidence interval; F, false equation; fMRI, functional MRI; MNI, Montreal Neurological Institute; MRI, magnetic resonance imaging; NA, not available; PW, phonologically and orthographically unfamiliar forms; sMRI, structural brain MRI; W, orthographic familiar forms of Icelandic nouns.

a *P*-values marked with ^a^ are corrected with respect to a region of interest (the left occipito-temporal lobe and the left parietal lobe).

All the brain regions highlighted in Figures 1, 2, 3 are listed here. The sample sizes are *n*=707 for sMRI, *n*=284 for the fMRI word paradigm and *n*=110 for the fMRI multiplication paradigm. The effects are calculated as (carrier status effect−mean)/|mean|.
