# Supplementary material for: 15q11.2 CNV affects cognitive, structural and functional correlates of dyslexia and dyscalculia
Source: Transl Psychiatry. 2017 Apr 25;7(4):e1109–. doi: 10.1038/tp.2017.77 (PMC5416713; doi:10.1038/tp.2017.77)

**Supplementary Material for Ulfarsson et al. 15q11.2 CNV affects cognitive, structural and functional correlates of dyslexia and dyscalculia**

**Supplementary Table 1 | The population characteristics for the subjects included in the MRI imaging experiments.** Age is reported at the time of scanning.

| **sMRI** | **15q11.2 del (n = 51)** | **PopCtrl (n = 552)** | **15q11.2 dup (n = 104)** |
| --- | --- | --- | --- |
| **Age (mean ± std)** | 42.8 ± 13.0 | 46.1 ± 11.5 | 45.6 ± 12.1 |
| **Gender (male, female)** | 24, 27 | 210, 342 | 39, 65 |
| **fMRI: Word experiment** | **15q11.2 del (n = 29)** | **PopCtrl (n = 189)** | **15q11.2 dup (n = 66 )** |
| **Age (mean ± std)** | 42.5 ± 12.7 | 44.6 ± 12.5 | 45.8 ± 11.7 |
| **Gender (male, female)** | 13, 16 | 77, 112 | 26, 40 |
| **fMRI: Word experiment (long version)** | **15q11.2.del (n = 16)** | **PopCtrl (n = 66)** | **15q11.2 dup (n = 56)** |
| **Age (mean ± std)** | 44.7 ± 12.0 | 45.4 ± 10.6 | 46.4 ± 11.6 |
| **Gender (male, female)** | 6, 10 | 28, 38 | 21, 35 |
| **Word experiment**  **(short version)** | **15q11.2.del (n = 13)** | **PopCtrl (n = 123)** | **15q11.2 dup (n = 10)** |
| **Age (mean ± std)** | 39.8 ± 13.6 | 44.1 ± 13.4 | 42.2 ± 12.2 |
| **Gender (male, female)** | 7, 6 | 49, 74 | 5, 5 |
| **fMRI: Multiplication experiment** | **15q11.2 del (n = 18)** | **PopCtrl (n = 40)** | **15q11.2 dup (n = 52 )** |
| **Age (mean ± std)** | 44.6 ± 12.9 | 45.7 ± 8.8 | 47.3 ± 11.7 |
| **Gender (male,female)** | 10, 8 | 17, 23 | 22, 30 |

**Supplementary Table 2 | The sample size for the cognitive tests and questionnaire in Figures a and b.**

|  | **VIQ** | **PIQ** | **LM** | **LF** | **CF** | **Stroop** | **TMT** | **Pers err** | **SWM** | **RVIP** | **TMT-A** | **Str-bl** | **GAF** | **ARHQ** | **AMHQ** |
| --- | --- | --- | --- | --- | --- | --- | --- | --- | --- | --- | --- | --- | --- | --- | --- |
| **DEL** | **67** | **67** | **71** | **71** | **71** | **71** | **70** | **66** | **65** | **61** | **70** | **71** | **71** | **55** | **55** |
| **DLX&DC** | **41** | **41** | **41** | **41** | **41** | **41** | **40** | **40** | **40** | **40** | **40** | **41** | **42** | **42** | **42** |
| **DLXonly** | **80** | **80** | **80** | **80** | **80** | **79** | **80** | **77** | **79** | **74** | **80** | **80** | **78** | **80** | **80** |
| **DConly** | **69** | **69** | **68** | **67** | **67** | **67** | **67** | **66** | **67** | **66** | **67** | **67** | **69** | **69** | **69** |
| **NoCNV w/o learning difficulties** | **450** | **450** | **451** | **450** | **450** | **450** | **448** | **438** | **444** | **416** | **448** | **450** | **443** | **452** | **452** |

**Supplementary Table 3 | Carrier status versus learning difficulty subgroups.**

|  | **w/o learning difficulties** | **DLXonly** | **DConly** | **DLX&DC** | **DLX** | **DC** |
| --- | --- | --- | --- | --- | --- | --- |
| **15q11.2 deletion** | 22 | 10 | 10 | 13 | 23 | 23 |
| **NoCNV** | 452 | 80 | 69 | 42 | 123 | 111 |

**Supplementary Table 4 | Carrier status dependent gray matter volume change.** All the brain regions highlighted in Fig. 1 are listed here.

| **Gray Matter:** | **Hemisphere** | **MNI coordinates (x, y, z)** | **Effect (%)** | **P-value (corrected)** | **P-value (uncorrected)** | **Brodmann** |
| --- | --- | --- | --- | --- | --- | --- |
| Fusiform Gyrus | Left | (-35, -36, -15) | +3.0 | 0.045 | 7.99 x 10^-6^ | BA37 |
| Intraparietal Sulcus | Left | (-20,- 49, 39) | +4.2 | 0.091 | 1.89 x 10^-5^ | BA40 |
| Inferior Frontal Orbital | Right | (38, 45, -23) | +3.3 | 0.146 | 3.43 x 10^-5^ | BA47 |
| Posterior Cingulate | Left | (-10, -12, 30) | +2.6 | 0.201 | 5.25 x 10^-5^ | BA23 |
| Cerebelum 8 | Right | (24, -63, -44) | +2.7 | 0.372 | 1.28 x 10^-4^ |  |
| Inferior Frontal Orbital | Left | (-39, -33, -14) | +2.8 | 0.468 | 1.86 x 10^-4^ | BA47 |
| Cerebelum 8 | Left | (-9, -63, -30) | +2.1 | 0.683 | 3.96 x 10^-4^ |  |
| Superior Temporal | Right | (39, -34, 4) | +1.8 | 0.694 | 4.12 x 10^-4^ | BA37 |
| Amygdala | Right | (16, 2, -15) | +1.0 | 0.676 | 5.28 x 10^-4^ |  |
| Hippocampus | Right | (27, -10, -20) | +1.8 | 0.808 | 6.34 x 10^-4^ |  |
| Superior Occipital | Left | (-22, -78, 24) | -4.8 | 0.016 | 2.39 x 10^-6^ | BA19 |
| Superior Frontal | Right | (20, 30, 52) | -5.0 | 0.016 | 7.29 x 10^-6^ | BA8 |
| Superior Occipital | Right | (26, -67, 28) | -4.4 | 0.236 | 6.53 x 10^-5^ | BA19 |
| Postcentral | Left | (-46, -10, 28) | -3.8 | 0.276 | 8.27 x 10^-5^ | BA4 |
| Thalamus | Left | (-15, -18, 15) | +4.3 | 0.434 | 8.87 x 10^-5^ |  |
| Cuneus | Left | (-4, -70, 25) | +2.5 | 0.615 | 1.49 x 10^-4^ |  |
| Temporal Inferior | Right | (51, -54, -9) | +2.7 | 0.672 | 1.64 x 10^-4^ | BA37 |
| Putamen | Right | (30, 0, 10) | +2.4 | 0.715 | 3.13 x 10^-4^ |  |

**Supplementary Table 5 | Carrier status dependent white matter volume change.** All the brain regions highlighted in Fig. 2 are listed here.

| **White Matter** | **Hemisphere** | **MNI Coordinates (x, y, z)** | **Effect (%)** | **P-value**  **(corrected)** | **P-value (uncorrected)** |
| --- | --- | --- | --- | --- | --- |
| Cerebelum Cruz 1 | Right | (28, -72, -32) | +7.7 | 6.84 x 10^-5^ | 1.13 x 10^-8^ |
| Paracentral Lobule | Right | (10, -30, 54) | +4.6 | 6.93 x 10^-4^ | 1.45 x 10^-7^ |
| Superior Temporal | Left | (-52, -12, 13) | +5.0 | 1.94 x 10^-3^ | 3.11 x 10^-7^ |
| Fusiform | Left | (-30, -46, -15) | +4.6 | 0.074 | 3.09 x 10^-5^ |
| Precentral | Left | (62, 2, 25) | +5.0 | 0.150 | 7.54 x 10^-5^ |
| Supramarginal | Left | (-58, -37, 24) | +5.1 | 0.224 | 1.13 x 10^-4^ |
| Frontal Mid Orbital | Right | (33, 40, -17) | +3.2 | 0.373 | 2.74 x 10^-4^ |
| Frontal Mid Orbital | Left | (-42, 27, -15) | +4.5 | 0.374 | 2.75 x 10^-4^ |
| Supramarginal | Right | (56, -24, 39) | +3.9 | 0.447 | 3.69 x 10^-4^ |
| Paracentral Lobule | Left | (-14, -34, 73) | +4.5 | 0.526 | 4.95 x 10^-4^ |
| Precuneus | Left | (-10, -58, 52) | +3.5 | 0.634 | 7.27 x 10^-4^ |
| Anterior Corpus Callosum | N/A | (4, 0, 22) | -4.6 | 6.84 x 10^-4^ | 1.43 x 10^-7^ |
| Amygdala | Right | (26, 2, -17) | -4.7 | 5.57 x 10^-3^ | 1.54 x 10^-6^ |
| Amygdala | Left | (-27, -3, -14) | -3.8 | 0.063 | 2.55 x 10^-5^ |
| Superior Frontal | Right | (22, 24, 52) | -4.8 | 0.446 | 3.69 x 10^-4^ |

**
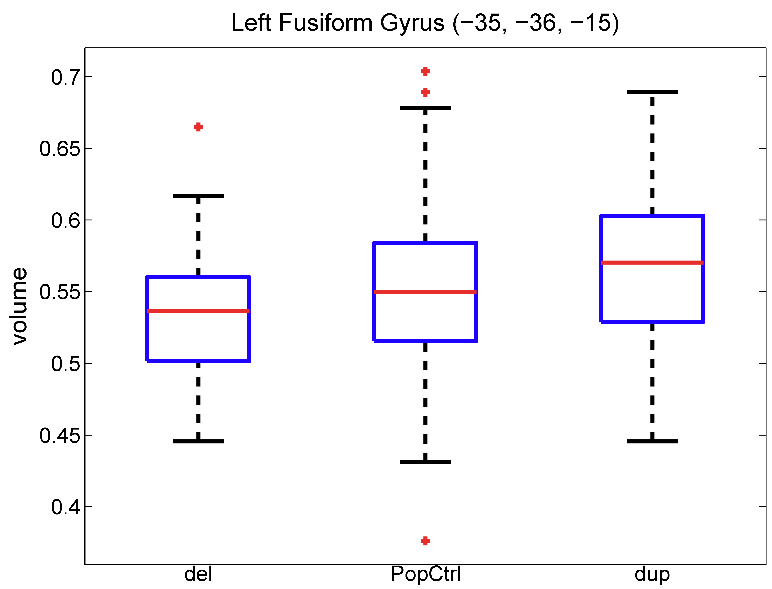
**
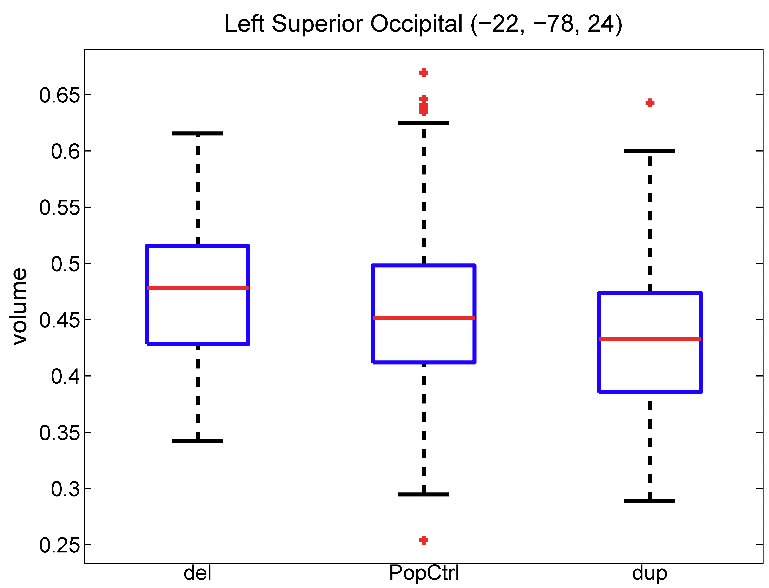


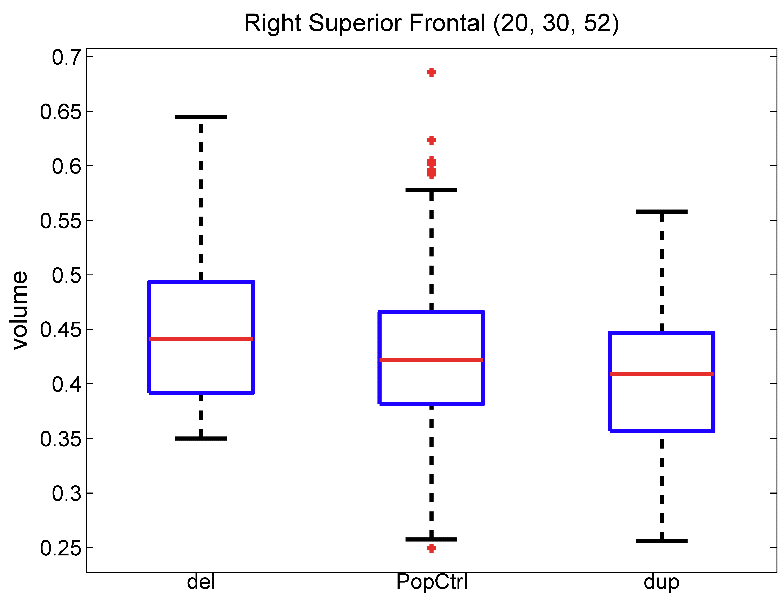


**Supplementary Figure 1 | Boxplots showing the smoothed modulated data at the peaks of maximal GM volume change for DEL, NoCNV, and DUP.**

**
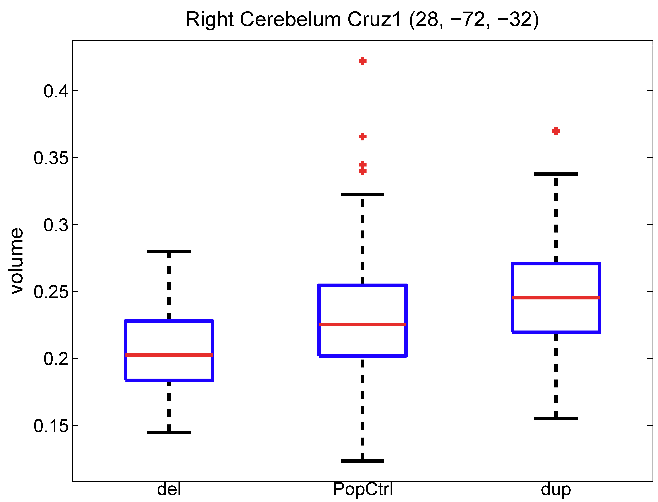

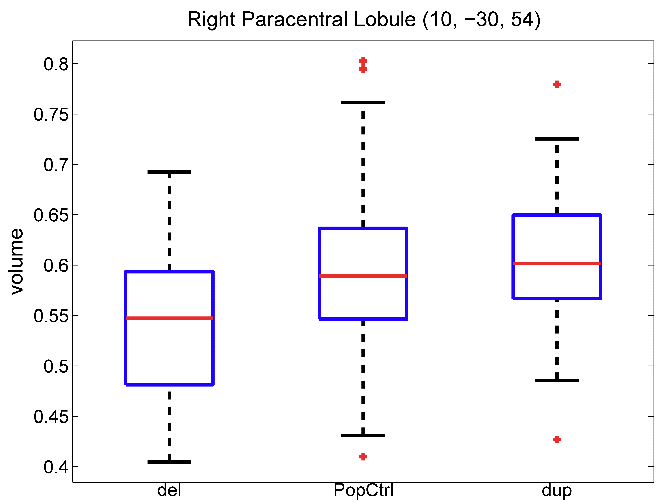

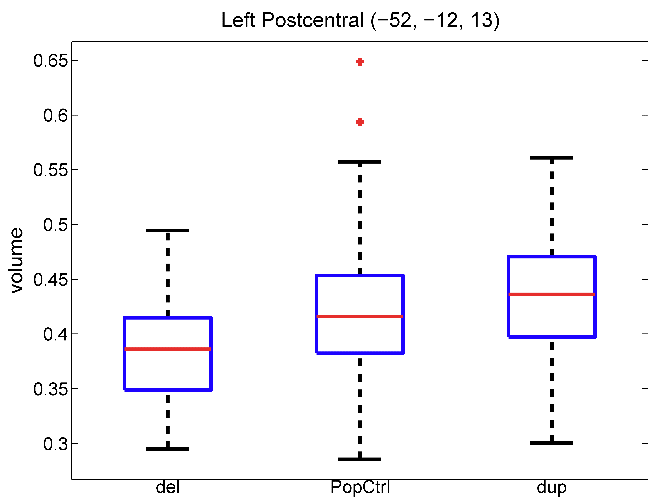

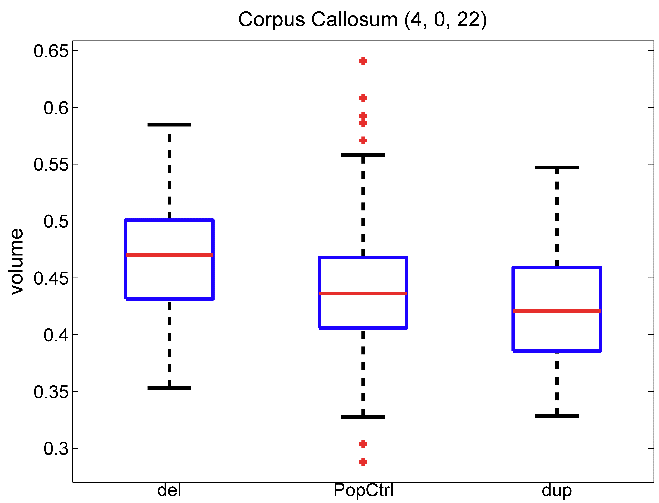

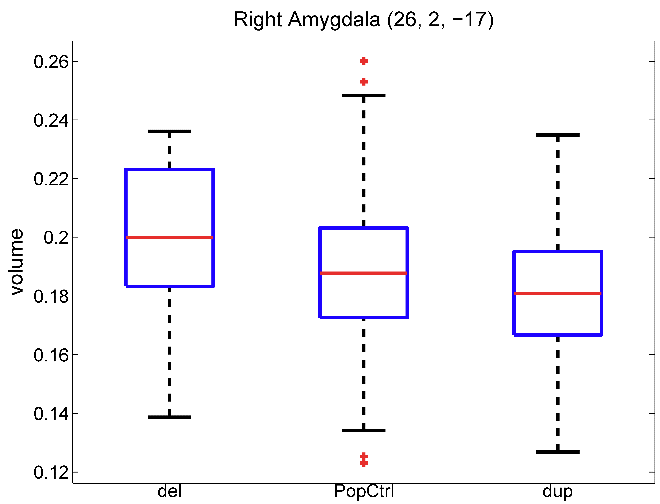
**

**Supplementary Figure 2 | Boxplots showing the smoothed modulated data at the peaks of maximal WM volume change for DEL, NoCNV, and DUP.**


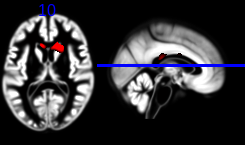


**Supplementary Figure 3 | An axial slice (left), z = 10, showing the location (MNI = (15, 22, 10), *P* = 0.012 (corrected), right caudate nucleus) of significant interaction between the carrier status (deletion, PopCtrl, duplication) of the gender and dosage. The right image shows the location of the axial slice on a sagittal view.**

**
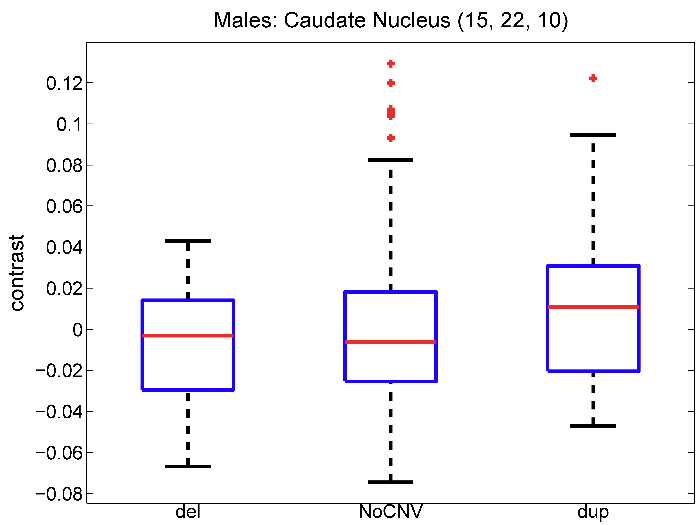

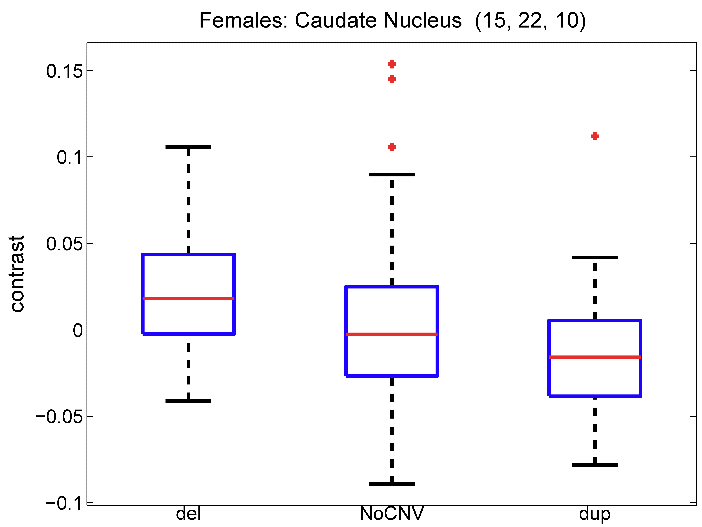
**

**Supplementary Figure 4 | Boxplots showing the smoothed modulated data at the peaks of gender and carrier status interaction.**


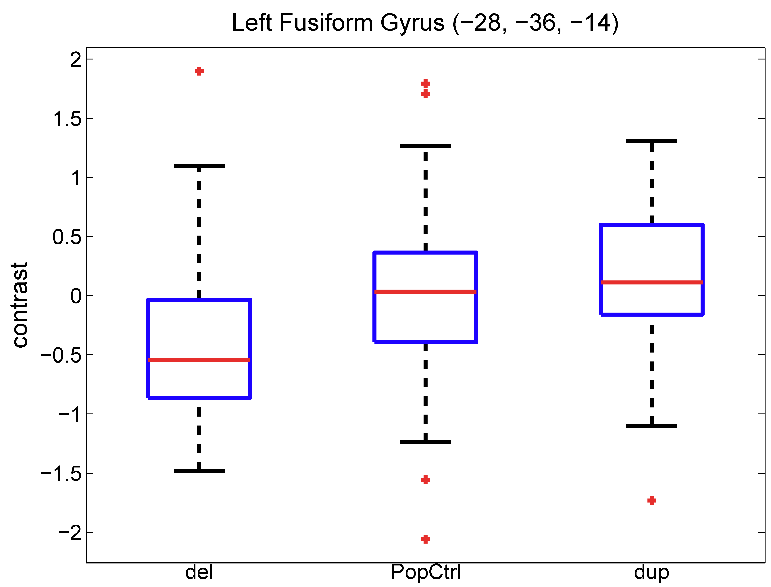

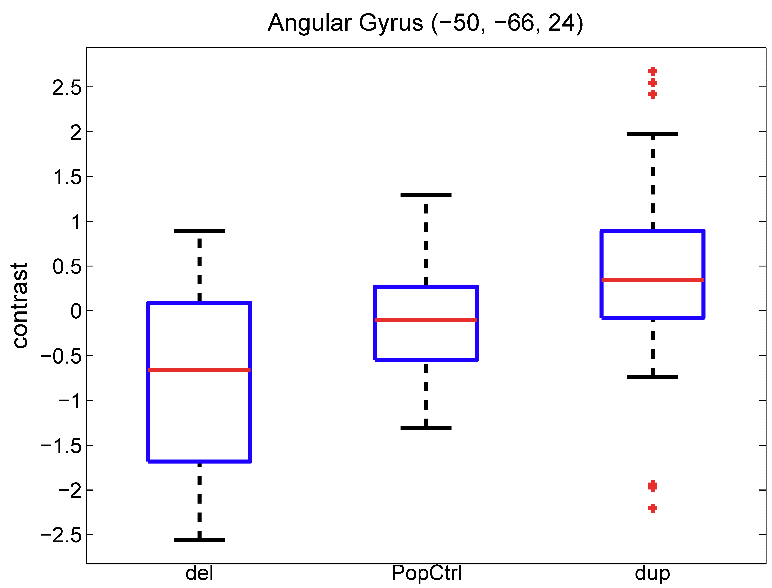


**Supplementary Figure 5 | Boxplots showing the fMRI data at the peaks of maximal activation change for DEL, PopCtrl, and DUP.**

**Supplementary Table 6 | The correlation matrix containing the Pearson’s correlations between each of the cognitive tests/questionnaire score in Figure 1 and the brain imaging phenotype in Table 1 for the NoCNV group.** Each correlation was tested for equality with zero, i.e., the null hypothesis was that the true correlation was equal to zero. Correlations with *P*-values less than 0.05/300, where 300 is the number of correlations in the upper diagonal of the matrix, are in boldface font.


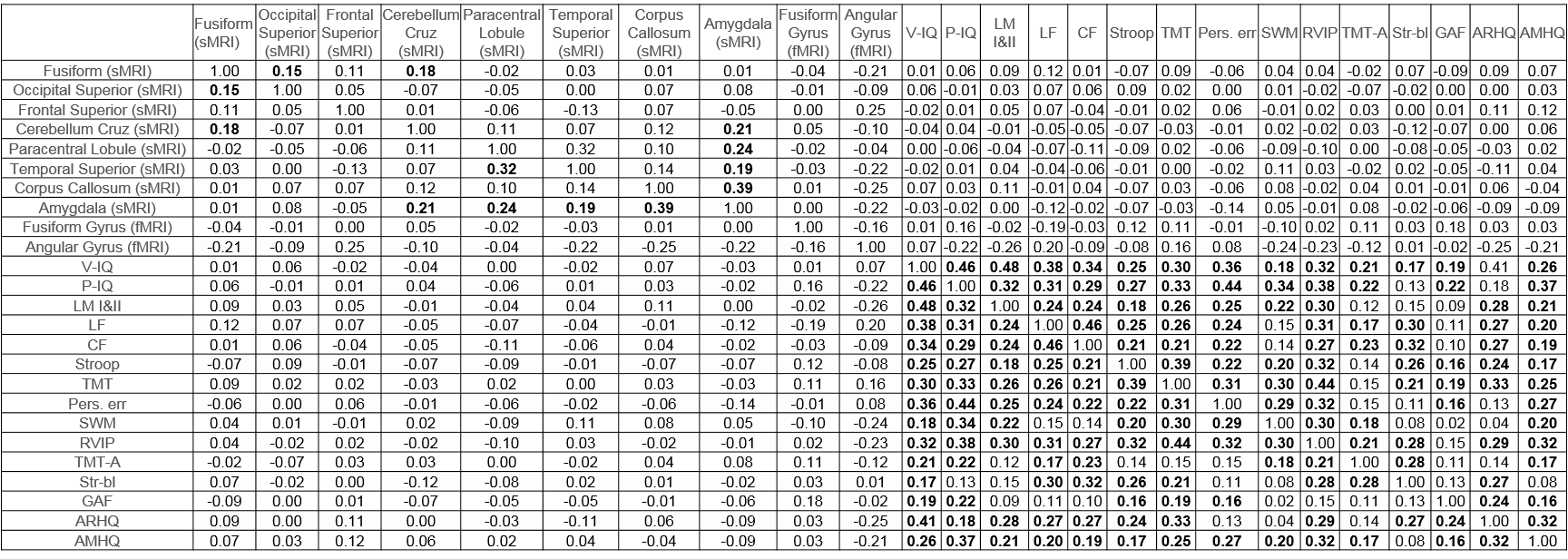


**Supplementary Table 7 | The sample sizes for the correlation in Supplementary Table 6.**


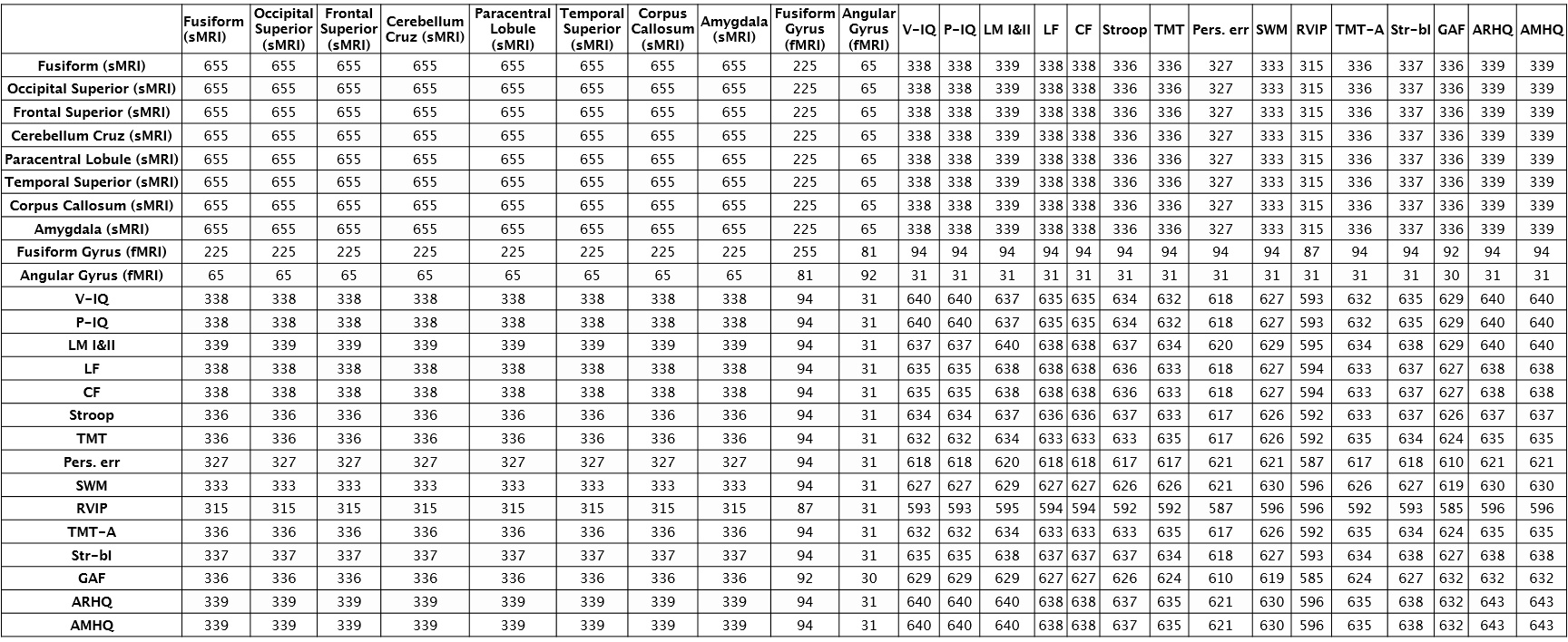


**Supplementary Table 8 | The correlation matrix containing the Pearson’s correlations between each of the cognitive tests/questionnaire score in Figure 1 and the brain imaging phenotype in Table 1 for the DEL group.** Each correlation was tested for equality with zero, i.e., the null hypothesis was that the true correlation was equal to zero. Correlations with *P*-values less than 0.05/300, where 300 is the number of correlations in the upper diagonal of the matrix, are in boldface font.


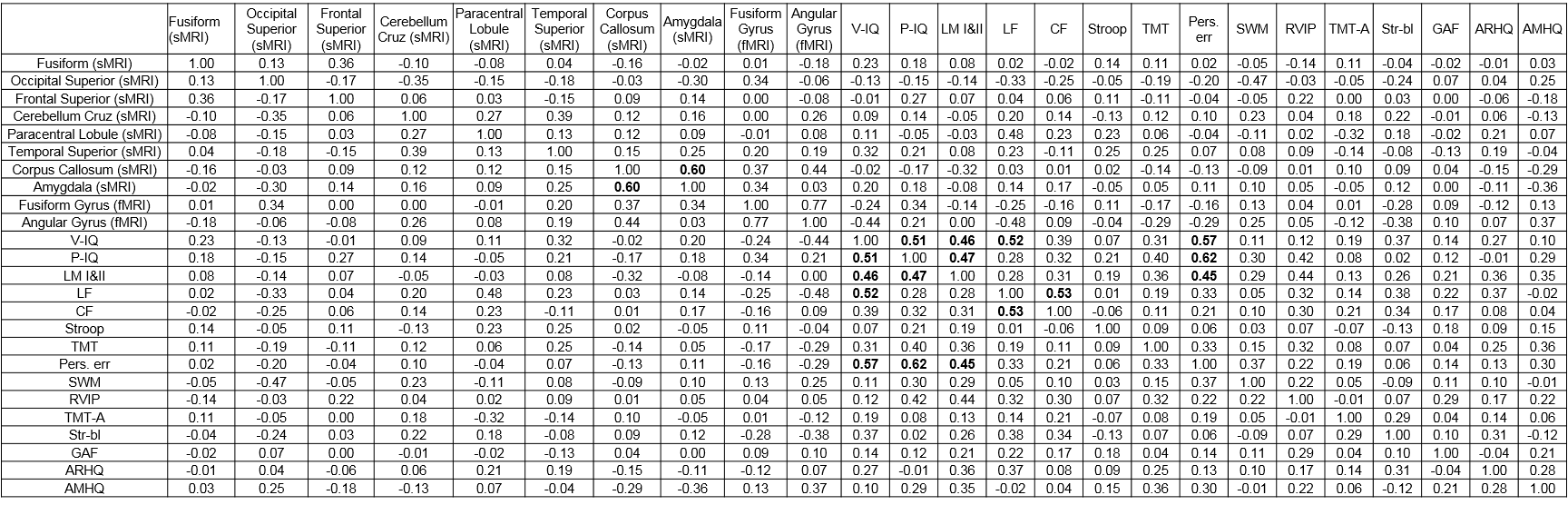


**Supplementary Table 9 | The sample sizes for the correlation in Supplementary Table 8.**


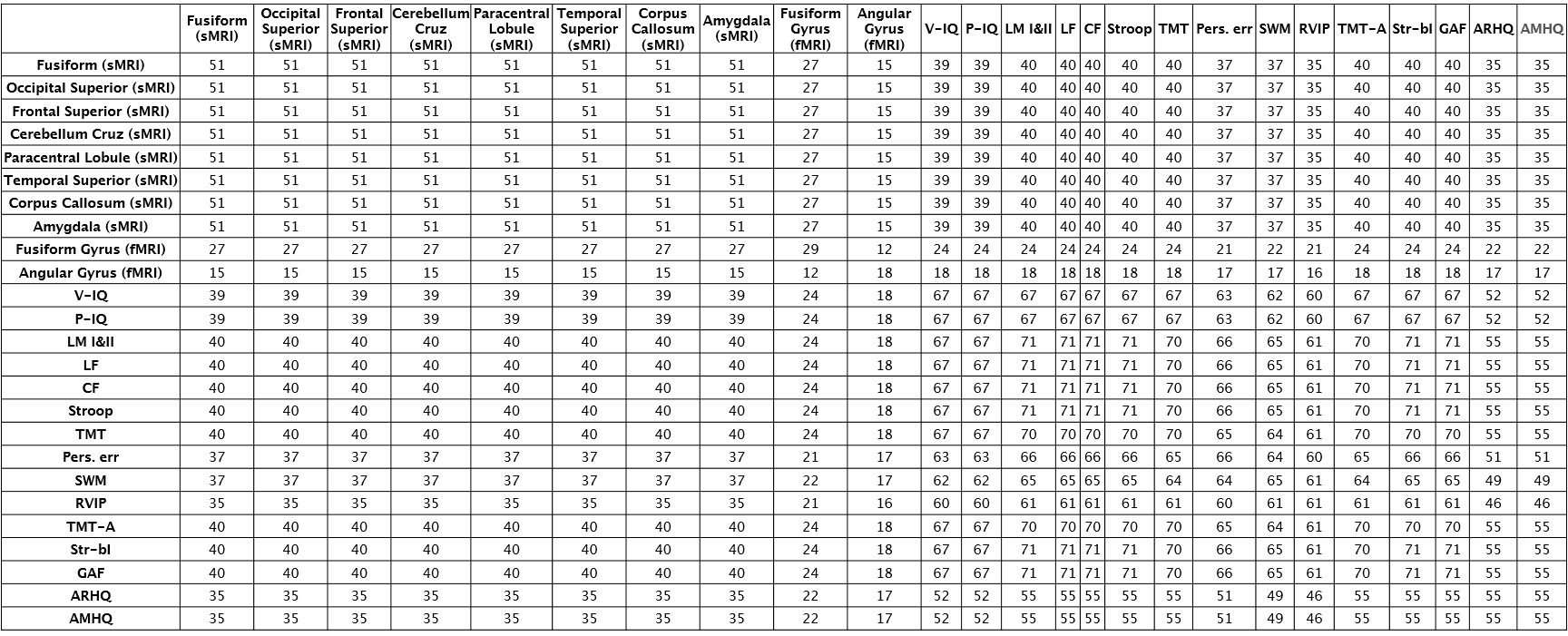

Supplement: Supplementary Information [file tp201777x1.docx]
